# Psychometric Testing of the BRI Unmet Need Instrument: A Comprehensive Measure of Dementia Caregivers' Needs

**DOI:** 10.1093/geroni/igab046.1253

**Published:** 2021-12-17

**Authors:** Morgan Minyo, David Bass, Kate McCarthy, Katherine Judge

**Affiliations:** 1 Cleveland State University, Cleveland, Ohio, United States; 2 Benjamin Rose Institute on Aging, Cleveland, Ohio, United States; 3 Benjamin Rose Institute on Aging, Benjamin Rose Institute on Aging, Ohio, United States

## Abstract

Compared to non-dementia caregivers, family/friend caregivers of individuals with dementia experience more negative caregiving consequences. One reason is the myriad of negatively impacted life domains including: managing symptoms; family communication; financial and legal matters; and finding and coordinating services. Few psychometrically tested measures exist for assessing the range of potential unmet needs of dementia caregivers. Such a measure would describe the frequency and correlates of unmet needs and provide a key outcome for intervention research. This study tested the psychometric properties of a comprehensive measure of unmet needs, the BRI Unmet Need Instrument. Data from 192 family/friend dementia caregivers was used to test reliability and four validity types. Results showed total unmet needs, as well as its nine subscales, had good reliability (Cronbach’s alpha .70 - .95). Discriminant validity was confirmed through factor analyses of the 45 unmet needs and items in measures of depression and care-related strain. Unmet need items loaded on separate factors that were deemed acceptable (.72-.38). Predictive validity was assessed by the association with depression, which was significant and an acceptable range (r = .22, p < .01). Convergent validity was confirmed by significant associations with three caregiver strain measures, mastery (r = .40, p <.01), emotional strain (r = .19, p < .01), and relationship strain (r = .15, p <.05). Good structural validity for nine predetermined unmet needs subscales was found using principal component analysis (loadings = .82-.39). Results suggest the BRI Unmet Needs Instrument is a ready-to-use, reliable and valid comprehensive measure.

